# Tribotronic control of an ionic boundary layer in operando extends the limits of lubrication

**DOI:** 10.1038/s41598-022-22504-6

**Published:** 2022-11-28

**Authors:** Akepati Bhaskar Reddy, Georgia A. Pilkington, Mark W. Rutland, Sergei Glavatskih

**Affiliations:** 1 System and Component Design, Department of Machine Design, KTH Royal Institute of Technology, 100 44 Stockholm, Sweden; 2 Division of Surface Chemistry and Corrosion Science, Department of Chemistry, KTH Royal Institute of Technology, 100 44 Stockholm, Sweden; 3 Laboratoire de Tribologie et de Dynamique des Systèmes, Ecole Centrale de Lyon, 69134 Ecully, France; 4 School of Chemistry, University of New South Wales, Sydney, NSW 2052 Australia; 5 Department of Electromechanical, Systems and Metal Engineering, Ghent University, 9052 Ghent, Belgium

**Keywords:** Mechanical engineering, Surface assembly, Chemical physics, Molecular self-assembly

## Abstract

The effect of electric potential on the lubrication of a non-halogenated phosphonium orthoborate ionic liquid used as an additive in a biodegradable oil was studied. An in-house tribotronic system was built around an instrument designed to measure lubricant film thickness between a rolling steel ball and a rotating silica-coated glass disc. The application of an electric field between the steel ball and a set of customized counter-electrodes clearly induced changes in the thickness of the lubricant film: a marked decrease at negative potentials and an increase at positive potentials. Complementary neutron reflectivity studies demonstrated the intrinsic electroresponsivity of the adsorbate: this was performed on a gold-coated silicon block and made possible in the same lubricant system by deuterating the oil. The results indicate that the anions, acting as anchors for the adsorbed film on the steel surface, are instrumental in the formation of thick and robust lubricating ionic boundary films. The application of a high positive potential, outside the electrochemical window, resulted in an enormous boost to film thickness, implicating the formation of ionic multi-layers and demonstrating the plausibility of remote control of failing contacts in inaccessible machinery, such as offshore wind and wave power installations.

## Introduction

The energy consumed to overcome friction and to address wear-related failures in machinery, contribute significantly to global energy consumption (estimated 23% in 2017)^1^. Tribological research focused on developing new surfaces, materials, and lubrication technologies aimed at reducing friction and wear in mechanical components, has been predicted to have massive potential for reducing energy consumption and emissions^1,2^. From a sustainable lubrication perspective, most of the research is directed towards developing environmentally-acceptable lubricants that aid in improving machine efficiency and reliability^3^. As modern machines are becoming increasingly compact (for increased power density) and electrified, the functional demands on the lubrication systems are increasing. This is leading to novel alternative lubrication concepts, such as tribotronics.

Tribotronics is a fusion of tribology and electronics, aimed at enhancing machine efficiency and durability through in-situ control of loss outputs, such as friction and wear^4^. A tribotronic system includes a tribological contact, sensors (monitoring friction, vibration, temperature, etc.), actuators, and a control unit^4^. Ionic liquids (ILs), defined as organic salts with melting points below a certain nominal temperature (usually 100$^\circ$C)^5^, have emerged as viable candidates for tribotronic actuation systems, because of their ionic nature. ILs also exhibit the ability to undergo changes to their ion dynamics under the influence of external factors like temperature, pressure and electric field^6^.

Good lubrication properties have been demonstrated by ILs as neat lubricants^7–13^, in some cases outperforming fully formulated lubrication oils^10,11,14^. Research efforts have also been made to synthesize and tribologically evaluate oil-soluble ILs for application as lubricant additives^15–24^. Some of these ILs have shown anti-wear performance comparable to ZDDP^17^, and also synergistic performance when used together^25^. Surface activity of ILs towards charged surfaces, resulting in the formation of ordered boundary films has been demonstrated^6,26–30^. These non-sacrificial films have been shown to assist in reducing friction and maintaining contact separation^31,32^. Since most widely available ILs were intended for chemical applications, their structures include halogens that tend to form toxic and corrosive halides on hydrolysis^33–36^. To address this issue, boron- and phosphorus-based non-halogenated ILs have been designed for lubrication research^37–47^.

The molecular architecture of the ionic species and their degree of dissociation in the oil could govern the morphology of the ionic boundary layer^48^. ILs with a high degree of dissociation in the oil form more structured and robust ionic boundary layers, thereby enhancing the lubricity of the oil^48^. A wide range of scientific techniques has been implemented to evaluate the possibility of inducing changes to the interfacial layer through electric potential and thereby achieving tribotronic control. Neutron reflectivity (NR) experiments have shown response of the interfacial layers, formed on a gold electrode by trihexyltetradecylphosphonium bis(mandelato)borate (P-BMB) IL dispersed in polar solvents, to applied electric potential in terms of layer thickness and ionic composition^26,27^. A positive potential increased anion concentration and a negative potential increased cation concentration in the rather diffuse (with a significant number of solvent molecules) interfacial layer. For the same but pure IL, atomistic molecular dynamics simulations of ionic structuring on neutral and charged electrodes revealed surface charge induced changes in ionic concentration, as well as the molecular orientations in the interfacial layers^49^. Weighing of the surface charge on a gold electrode for the pure IL, 1-ethyl-3-methylimidazolium tris(pentafluoroethyl)trifluorophosphate, through electrochemical quartz crystal microbalance (QCM) has also revealed electric potential driven changes in the cation to anion ratio associated with the electrode surface^50^.

Normal force curve measurements using an atomic force microscope (AFM) have shown that the nature of the interfacial structure, induced by an electric potential, depends on the ionic architecture^51,52^. Small anions promote better packing at positive potentials^51^. The cationic structure with either imidazolium ring strongly bound to the surface or long alkyl chains (enhancing solvophobic interactions) have been shown to promote more efficient structuring at negative potentials^51,52^. AFM studies revealed that strong and efficient interfacial structure induced by electric potential resulted in low friction^27,53^. The impact of sliding speed on friction is reduced by this strong interfacial layer due to the formation of a well-defined sliding plane^53^. At high potentials, the formation of a multi-layer ionic structure to neutralize the surface potential has also been reported^54,55^. The intensity of electro-response of IL dispersed in a solvent is dependent on the bulk IL concentration, with negligible electro-response observed for trihexyl(tetradecyl)phosphonium bis(2,4,4-trimethylpentyl)phosphinate (P-BMPP) IL concentration less than 5 mol % in a model solvent - hexadecane^56^. NR studies have also shown that an increase in bulk IL concentration increased the ionic concentration in the interfacial layer, thereby enhancing the electro-response^27^. AFM studies have shown that the electric potential induced ion arrangement in the boundary layer, and the resulting frictional response, also vary with the nature of the substrate material^57^. Some ILs have been reported to dramatically change friction^58–60^, and even produce “super-lubricity”^59,60^ when an electric potential was applied. These findings have also been backed up by non-equilibrium molecular dynamics simulations of nano-scale IL films on charged surfaces^61–63^.

The AFM studies and the surface-sensitive analytical techniques like NR and QCM, evaluate the electro-response of ILs on smooth substrates such as gold, graphite, or mica. However, these results cannot be directly transferred to the macro-scale tribological contacts formed by rough surfaces^64^. Limited studies on macro-tribological contacts have demonstrated changes in IL frictional behavior with applied electric potential^65–67^. Permanent changes to the frictional response of a mixture of two ILs through the application of an electric impulse has also been reported^68^. However, these studies do not provide evidence for reversibility of tribological performance with change in potential bias, which is important for developing systems with in-situ tribological control.

Here we have studied the electroresponse of the lubricating film formed by a non-halogenated IL, trihexyl(tetradecyl) phosphonium bis(oxalato)borate (P-BOB), added to a biodegradable oil (2-ethylhexyl laurate: 2EHL), using an in-house developed tribotronic system. This IL, when used as a low concentration additive (1% wt.), forms thick and robust non-sacrificial adsorbed ionic boundary films, enhancing the lubricity of the oil^48^.

## Methods

### Oil and IL

**Figure 1:**
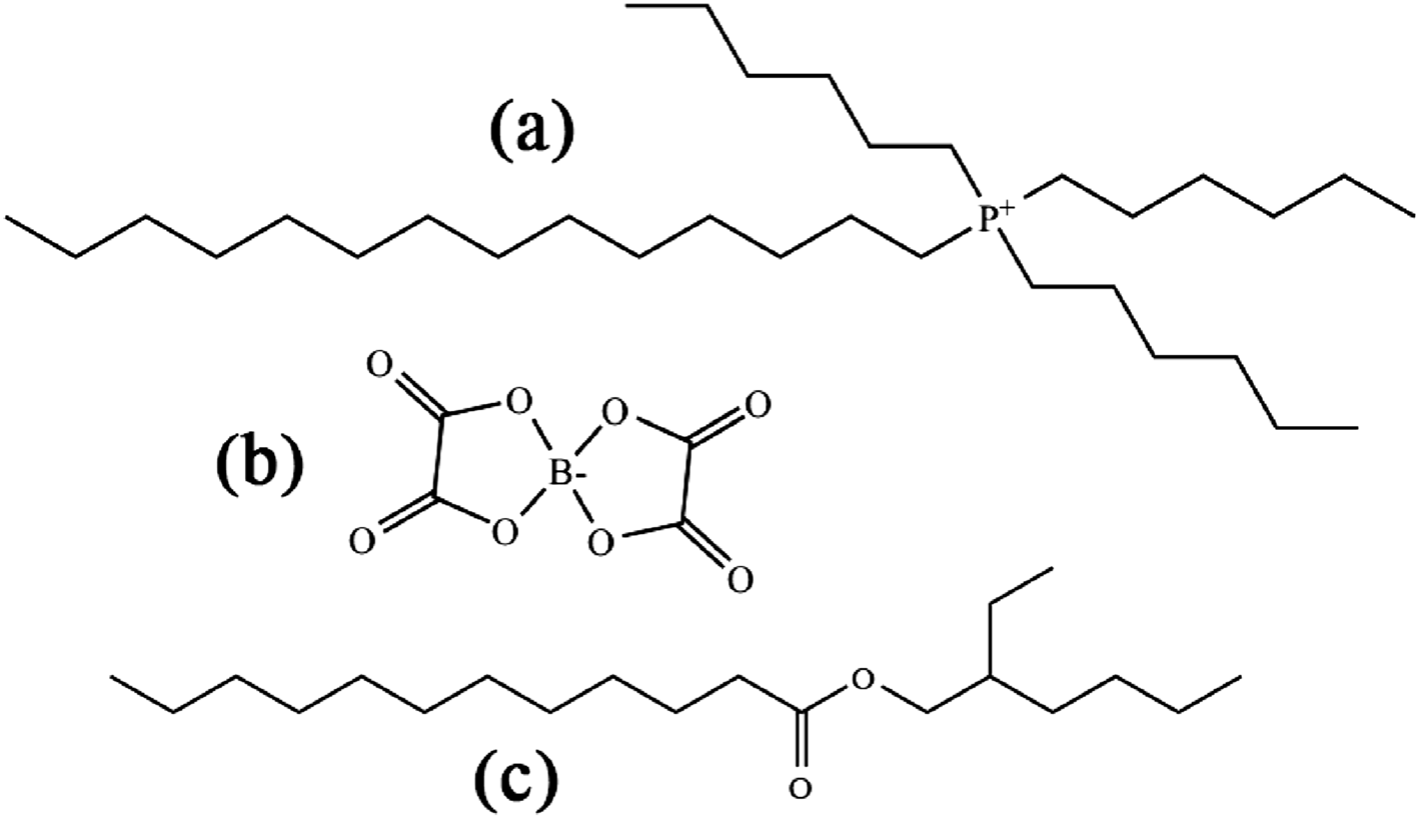
Trihexyl(tetradecyl)phospohnium cation (**a**); bis(oxalato)borate (BOB) anion (**b**); and base oil, 2-ethylhexyl laurate (2EHL) (**c**).

The molecular structures of the base oil and the IL used in this study are shown in Fig. 1. The base oil, 2-ethylhexyl laurate: 2EHL (Dehylub^®^ 4003, Emery Oleochemicals GmbH) is a readily biodegradable (91.7% biodegradation in a 28-day period^69^) synthetic ester with a low viscosity (4-6 cSt at 40$^\circ$C).

The IL used in this study was trihexyltetradecylphosphonium bis(oxalato)borate (P-BOB)^70^. P-BOB has been shown to form robust friction and wear reducing ionic boundary films when used as a low concentration (1% w/w) additive in 2EHL^48^. This study also showed that the IL is miscible in 2EHL at least to 10 % w/w concentration.

NR and nanotribology experiments showed that the composition and extent of the IL boundary layers is concentration dependent and distinct electroresponsive changes could be observed at high IL concentrations^27^. Therefore, an IL concentration of 5% w/w in 2EHL was implemented to obtain distinct electroresponsive effects. For preparing the ionic solution, the weighed components (2EHL and P-BOB) were combined and then shaken, followed by heating to 40$^\circ$C for 1 h and then ultrasonication for 10 min.

### Film thickness measurements

The central film thickness in the elastohydrodynamic (EHD) contact was measured using an EHD2 test rig from PCS Instruments, UK. EHD2 uses optical interferometry to measure the ultra-thin film thickness between a steel ball (3/4” diameter) and a glass disc coated with silica spacer layer with an uncertainty of ± 1 nm^71^. The ball and the disc are rotated individually to attain the required entrainment speed and slide-roll ratio (SRR).

**Figure 2:**
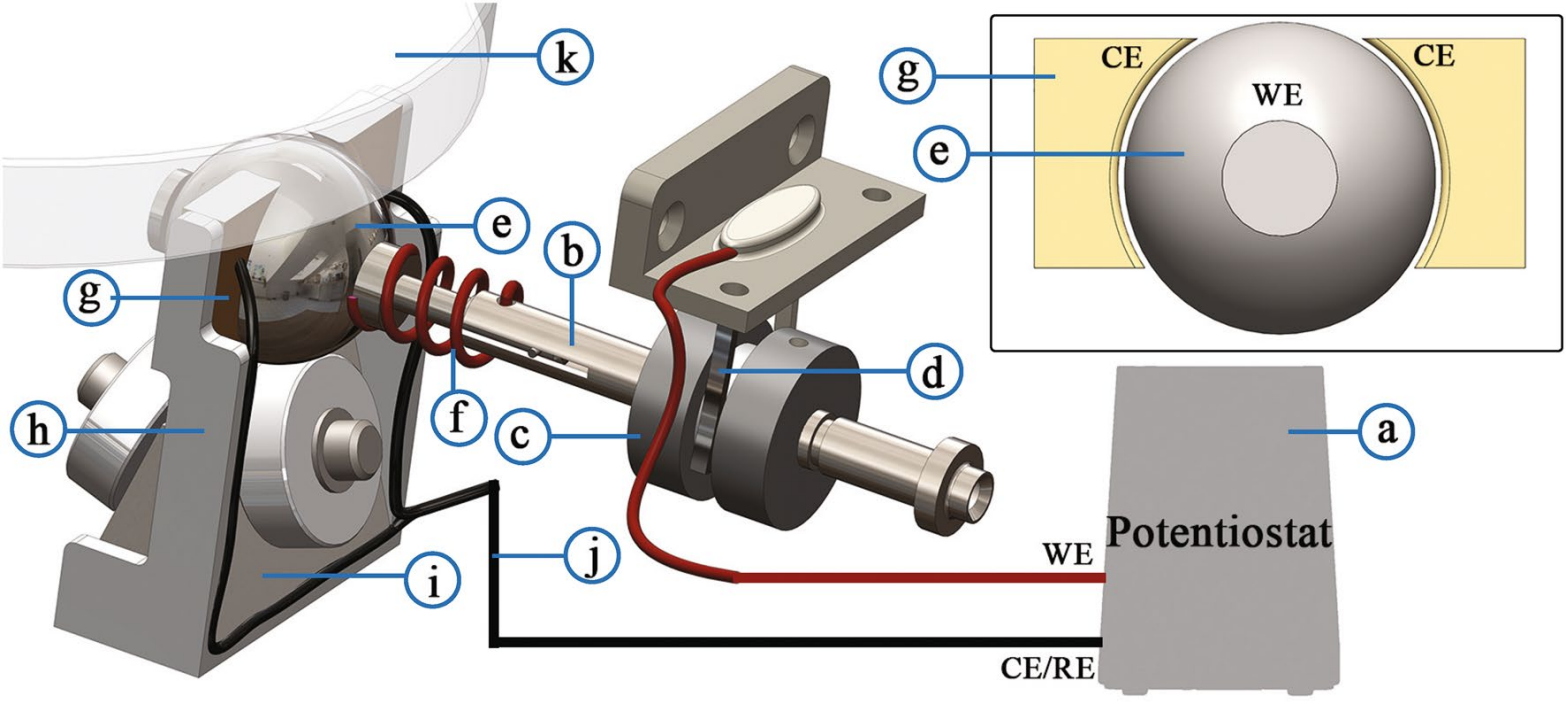
Schematic of modified EHD2 with a 2-electrode setup. The working electrode (WE) of the potentiostat (**a**) is connected to the ball drive shaft (**b**) through a slip ring body (**c**) and slip-ring brush (**d**). The drive shaft is further electrically connected to the ball (**e**) through a coiled wire (**f**). The drive shaft is electrically insulated form the rest of the equipment by the use of ceramic bearings (not shown). The counter electrodes (CE) for the setup are composed of two stainless steel blocks (**g**) on either side of the ball. The inner face of the blocks has a spherical face allowing a gap of approximately 0.5 mm between each CE and the ball surface (inset). These block are held in place by a frame (**h**) which is integrated into the ball carriage (**i**), which is composed of a 3-bearing geometry (ceramic bearings for electrical insulation) on which the ball rests. A strip of PTFE (not shown) electrically insulates the blocks from the frame. The blocks are connected to the potentiostat CE terminal using a set of wires (**j**). The ball carriage translates vertically to obtain the required contact load between the ball and the glass disc (**k**). (Solidworks 2019, Dassault Systèmes; available at https://www.solidworks.com/).

The EHD2 test rig was modified to incorporate a tribotronic system. The schematic of the setup modifications is shown in Fig. 2. The setup uses a 2-electrode setup, with the reference electrode (RE) and the counter electrode (CE) jumped. The steel ball was the working electrode (WE). The counter electrode was composed of two stainless steel (AISI 304) blocks positioned on either side of the ball. The blocks had a spherical inner surface, allowing for an electrode gap (between WE and CE) of about 0.5 mm. The blocks were positioned on a plane perpendicular to the ball axis, allowing for the ball contact track (tribo-track) to continuously pass through the electric field created between the WE and CE during rotation. The test solution was filled till the CE blocks were submerged. A Metrohm Autolab (PGSTAT204) potentiostat was used for applying electric potential between the electrodes. During the tests, the applied potential and current across the electrodes were continuously monitored.

Before each experiment, the test samples, glass disc and steel ball, were ultrasonicated in heptane and iso-propanol for 30 min each. The ball carriage was soaked in heptane and iso-propanol, successively. All the samples and accessories were rinsed with ethanol before drying. The spherical surfaces of the CE were polished using Kemet diamond paste (25 µm) to remove the organic and electrochemical residue deposited during the preceding test. Experiments were performed at pure rolling condition (0 % SRR) at 40$^\circ$C (Table 1).

**Table 1:** Test materials and conditions.

	Film thickness measurements		NR experiments
	Ball	Disc	Substrate
**Sample properties**			
Surface material	AISI 52100	Silica	Gold
Surface finish	Ra < 0.02 µm	Ra < 0.008 µm	Rq < 0.001 µm
Hardness	HV 800–920		
**Tribological conditions**			
Temperature	40$^\circ$ C		n.a.
Load	20 N		
Max. Hertzian pressure	0.5 GPa		
Hertzian contact radius	140 µm		
SRR	0% (pure rolling)		

### Cyclic voltametry

Cyclic voltammetry (CV) measurements were performed for the solution and electrode setup to establish a safe electrochemical window within which the effect of Faradaic events are minimized. For these measurements, fresh 2EHL + P-BOB solution and samples were used in the same tribotronic setup as described above. The current response was recorded while cycling the potential between -2 and 2 V, starting from OCP in anodic direction, with a scan rate of 100 mV/s.

### Neutron reflectance

Electrochemical neutron reflectivity (NR) measurements were performed at a gold working electrode at different potentials for 5% w/w solution of P-BOB in 2EHL to evaluate the interfacial structuring and its electroresponse. This technique utilizes the contrast between the scattering length densities (SLDs) of the bulk solution, ionic species, and the substrate for probing the composition and the thickness of ionic boundary film. The choice of gold as the substrate was dictated by the need for a homogeneous and almost atomically smooth surface, which cannot be easily achieved with, in order to capture minute changes in the boundary film. This experiment was performed at Australian Nuclear Science and Technology Organization (ANSTO), Australia. The experimental procedure, the construction of the electrochemical cell, and the data fitting methodology have been described elsewhere^26^. For highlighting any differences at the gold interface compared to the bulk, the IL mixture was contrast matched to the gold SLD ($4.56 \times 10^{-6}$ Å$^{-2}$) using appropriate ratio of hydrogenated 2EHL and perdeuterated 2EHL. A two layer interfacial model was found to provide the best fit to the reflectivity curves.

## Results and discussion

**Figure 3:**
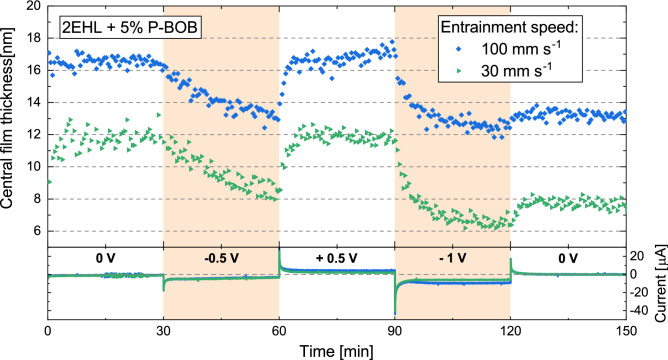
Electro-response of the central film thickness for 2EHL + 5% P-BOB solution at 100 and 30 mm s$^{-1}$ entrainment speeds. The applied potential was changed every 30 min. The bottom part of the figure shows the current traces corresponding to the applied potentials.

**Figure 4:**
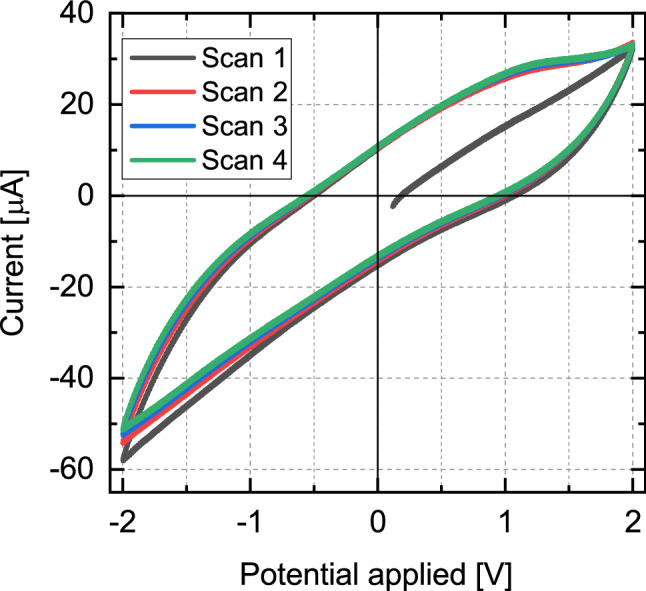
Four consecutive CV scans between + 2 and − 2 V, with the first scan starting at OCP (around + 0.1  V), for 2EHL + 5% P-BOB solution.

Tribotronic film thickness experiments were performed for 2EHL + 5% P-BOB solution using the experimental and electrochemical setup described in the “Methods” section. The film thickness was measured at a constant entrainment speed and load. Figure 3 shows electro-responsive film thickness plots for the solution at two different entrainment speeds (100 and 30 mm s^-1^). The bottom part of the figure depicts the current traces corresponding to the electric potentials applied for 30 min each. These applied potentials were within an electrochemical window of − 1 to + 0.5  V, established through cyclic voltammetry (CV). The CV scans (Fig. 4) show an oxidation peak at around + 1.25 V while being linear up to around + 0.75 V. Although a reduction peak is not achieved within the scanned potentials (− 2 to + 2 V), the slope of the curve increases sharply for potentials below − 1 V. Therefore, within the established electrochemical window the effects of Faradaic processes are expected to be minimal.

While the neat 2EHL oil does not exhibit any electroresponsivity (Supplementary Fig. S2), the film thickness for 2EHL + P-BOB solution responded strongly to the applied electric potential at both the entrainment speeds (Fig. 3). At 0 V, a consistent film thickness is observed. The magnitude of the film thickness corresponds to the level of stabilized film thickness for the open circuit (OC) condition (Supplementary Fig. S1), without the thick initial films. Given that the open-circuit potential (OCP) of the system is slightly positive ($\approx$ + 0.1 V), an application of 0  V should slightly change the adsorption and structuring of the boundary layer. This is confirmed by the small negative current measured during this step (an enlarged plot of the current trace during the 0 V step is shown in Supplementary Fig. S3). However, the absolute composition of the boundary film at this potential cannot be determined.

At negative potentials, an ionic exchange is expected to occur between the boundary film and the bulk, increasing the cation density in the boundary film. When such negative potentials were applied, a gradual decrease in film thickness was observed that appeared to depend on the magnitude of the negative potential. This suggests that the cation-rich boundary films do not adsorb efficiently, and hence cannot withstand the continuous rolling conditions. Additionally, the film thickness seems to gradually fall towards the initial film thickness level for 2EHL oil (Supplementary Fig. S1), supporting the hypothesis that the ionic boundary film gradually depletes. The film was easily restored by the application of positive potential, where the interfacial film is expected to be anion-rich. This shows that the anions are instrumental in defining the load-carrying capability of the ionic boundary film. Anions, with four surface-active carbonyl groups, act as anchors for forming strong adsorbed films at 0 V and positive potentials. In contrast, with negative potentials, the anion concentration in the boundary film decreases, resulting in a deprivation of the anchoring capability of the boundary film. A mild recovery and stabilization of film thickness are observed during the last 0 V step (120–150 min), hinting at a reorganization of the interfacial film to regain some of the load-carrying capacity. However, a stronger positive potential bias would be required for a more substantial film thickness recovery. The current traces also show a spike at the moment of potential change which then converges to a low, but finite, current value. This suggests a significant ionic mobilization and reorganization with the change in electric potential bias. It can also be observed that these film thickness data have a much smaller scatter compared to the open-circuit film thickness (Supplementary Fig. S1), suggesting a controlled and well-defined interfacial structuring. These results show in-situ controllability of lubricant film thickness through a variation in electric potentials within the electrochemical window of the oil+IL solution. Thereby, the contact separation can be controlled. Although these experiments are focused on modifying the adsorbed films on the steel ball surface, it is worth noting that the measured lubricating film also includes a boundary film adsorbed on the silica surface of the glass disc, the nature of which and the effects of charge accumulation on silica surface are unknown.

**Figure 5:**
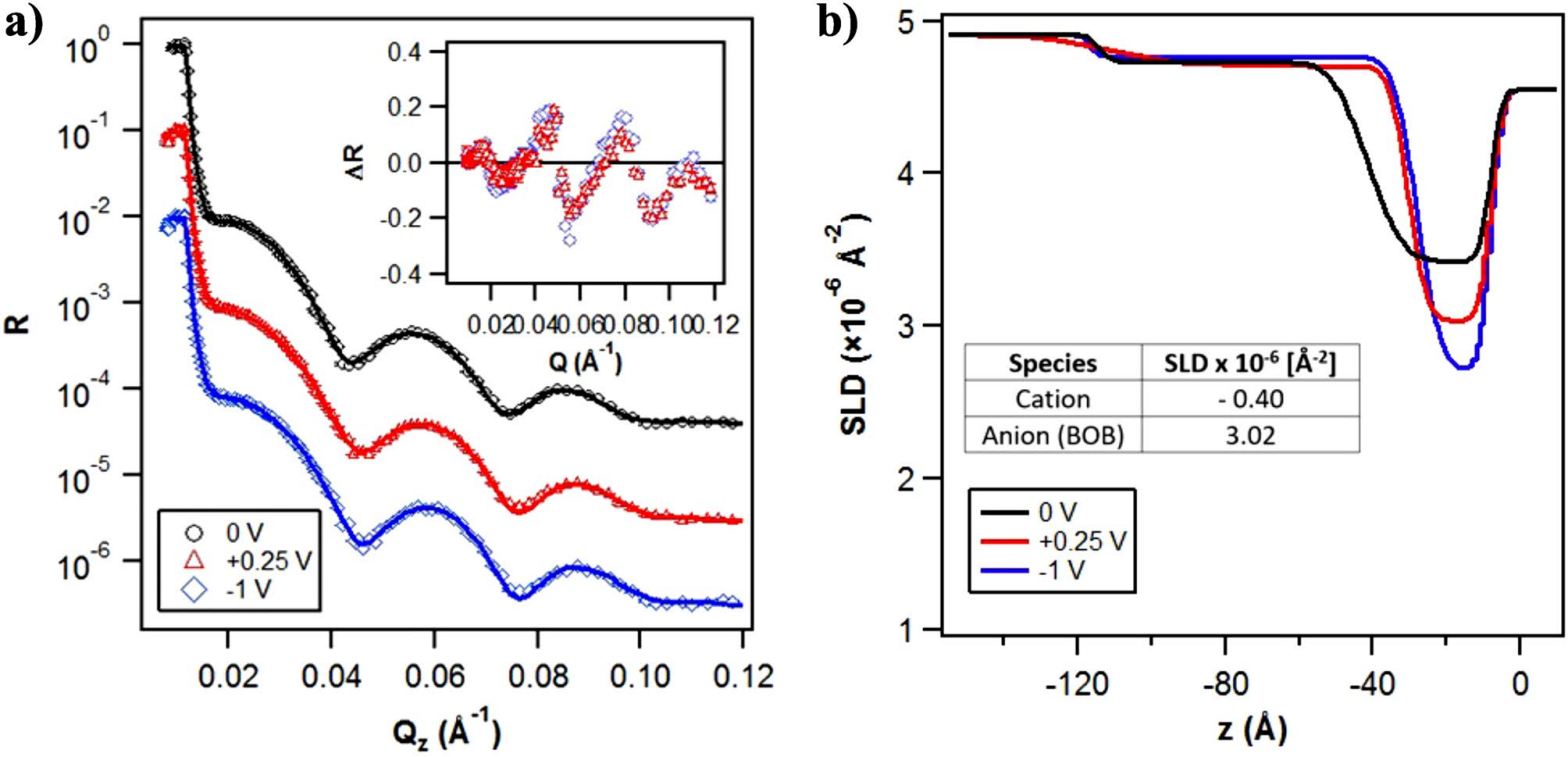
(**a**) NR reflectivity curves for contrast matched 2EHL + 5% P-BOB at different applied potentials. The curves are offset in the Y-axis for clarity. The symbols represent experimental data, whilst the solid lines are best-fits to the data. Inset: Asymmetry plot highlighting the changes in the non-zero potential NR curves with respect to the 0 V curve. (**b**) SLD profiles obtained from the best fits to the NR data (z = 0 at the gold/solution interface).

The electroresponsivity of the ionic boundary film has been tested through a neutron reflectance experiment. The gold surface with sub-nanometer roughness can be probed with neutrons, and the response to electric fields measured^26–28^. Figure 5a shows a neutron reflectance experiment in the same oil solution as the tribological experiments. The presence of the Kiessig fringes in the profile indicates a well-defined boundary layer on the surface with a different scattering length density (SLD) than the bulk liquid^26^. The fitted SLD profile (somewhat analogous to a refractive index for neutrons) of this ion-rich layer (Fig. 5b) reveals the layer thickness and composition. The best fit implies a distinct interfacial layer, with a second region distinguishable from the bulk but with a much lower ionic content. The application of an electric potential shows a measurable change in the interfacial layer, which becomes thinner and better defined compared to that at 0 V. The lower SLD of the interfacial layer for − 1 V, compared to that at + 0.25 V, indicates a relatively higher cation density (due to cation having much lower SLD than the anion). Since the substrate is different (the challenges of obtaining a steel surface with Å roughness and homogeneous composition over the necessary area of 25 cm$^2$ were deemed to be too great for this study), there is no expectation of an exact match of the behavior with that on the tribological surfaces. However, the broad features are highly reminiscent. Firstly, there is a clear formation of a well-defined ion-rich boundary layer on the nanometer scale. Secondly, the composition and thickness of the layer change in response to the electric potential.

**Figure 6:**
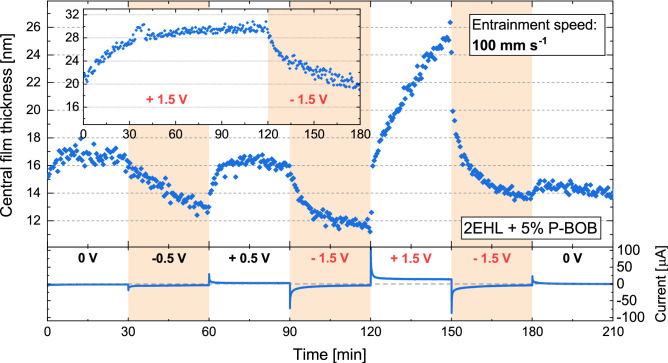
Electroresponse on central film thickness for 2EHL + 5% P-BOB solution with high voltages (− 1.5 and + 1.5 V: outside the electrochemical window) applied. The potential was changed every 30 min, with the first 2 potential changes being within the electrochemical window. The bottom part of the plot shows the current trace corresponding to the applied potential. The figure inset shows a snippet from the continuation of the same test where the high potentials were applied for a longer duration of time (+ 1.5 V for 2 h followed by − 1.5 V for 1 h).

Additional tests were conducted in the tribotronic EHD setup to determine the effect of potentials outside the established electrochemical window. Figure 6 presents a film thickness plot with high potentials included. The first two potential changes were within the electrochemical window. They were followed by the application of − 1.5 and + 1.5 V successively. A − 1.5 V potential gave a similar response to that of the “low” negative potentials. On switching to + 1.5 V (120 to 150 min), the film thickness increases dramatically (by about 14 nm) and doesn’t appear to reach steady-state within the 30 min potential application period. The “high” positive potential appears to trigger a massive ionic reorganization, resulting in multi-layer anion-rich interfacial structuring and leading to a very thick lubricating film. Notably, the film thickness is restored almost instantaneously on the application of higher electric potential, which is a prerequisite for a “red-button” approach to rescuing a tribological system. However, at this potential, the occurrence of Faradaic oxidation reactions on the WE and the resulting accumulation of oxidation products cannot be ruled out. The subsequent application of − 1.5 V decreases the film thickness, albeit to a slightly higher level compared to the earlier − 1.5 V step. This difference ($\approx$ 2 nm) could provide an estimate for the impact of Faradaic reaction during the preceding + 1.5 V step, suggesting that any reaction products have only a minor contribution towards the overall film thickness increase during the 30 min of + 1.5 V applied potential. To evaluate the steady-state film thickness resulting from the application of “high” potentials, the test was continued with these potentials applied for a longer duration (+ 1.5 V for 2 h followed by − 1.5 V for 1 h). A snippet of film thickness data from this extended test is shown in Fig. 6 Inset. Here, it was observed that the film thickness due to + 1.5 V stabilized at a very high level ($\approx$ 30 nm). The subsequently applied negative potential, while decreasing the film thickness, appears to converge to a much higher level ($\approx$ 20 nm) compared to the previous − 1.5 V step ($\approx$ 14 nm). The difference in the film thickness level of the two − 1.5 V steps (20 nm − 14 nm = 6 nm) also correlates well with the difference in the film thickness levels during the two preceding + 1.5 V steps (30 nm − 25 nm = 5 nm). This suggests that with prolonged exposure to a “high” positive potential, the non-reversible contribution to the film thickness from the Faradaic oxidation reactions becomes more significant. These results provide a promising approach to designing tribotronic systems using ILs. Moreover, deteriorating lubrication can potentially be rescued through a short application of electric potential, triggering the formation of the protective multilayer films on the surface.

## Conclusions

The self-assembly properties of some ILs as well as the ability to modify the interfacial layers via external electrical actuation makes ILs an attractive option for developing tribotronic systems. Through an electro-responsivity study of a macro-scale tribological contact, lubricated by a biodegradable oil with a phosphonium-orthoborate IL as an additive, we have demonstrated the potential to externally influence the lubricant film thickness, and hence the contact separation. Upon applying electric potential, significant systematic changes were observed in the thickness of the lubricating film, consisting of ionic boundary and elastohydrodynamic films. It was found that the cation-rich boundary film at negative potential is weakly adsorbed to the surface, resulting in a gradually decreasing film thickness. The film thickness was easily restored at a positive potential, indicating that the polar carboxylic groups on the anion serve as anchors in the formation of a strongly adsorbed ionic film. The NR experiment on gold revealed ionic enhancement at the interface and changes in the relative cation/anion densities upon application of electric potential, supporting the contention that the electric field changes the surface conformation. While nanotribology has long validated the concept of tribotronics, these results demonstrate that the electroresponsivity of the IL interfacial structure can be implemented to macro-scale tribological contacts for *in operando* control of lubricity.

ILs generally have a wide electrochemical window which permits the concept of tribotronics. Within this window, significant changes over reasonable timescales can be achieved, as we have now clearly demonstrated. Nonetheless, by exceeding this electrochemical window, when applying larger, but still rather modest, voltages, dramatic changes can be induced which are also faster. Since the currents are low, any electrical damage is estimated to be negligible, particularly in comparison to the equivalent damage from poorly lubricated contacts. This discovery provides the possibility of condition monitoring-driven rescue of lubrication by the tribotronic system. On detection of deterioration of lubrication, a short burst of high electric potential, outside the electrochemical window, can be applied to form a thick, surface-protecting multilayer ionic film. Although a contribution to the film thickness from electrochemical reaction products cannot be completely ruled out, this may nonetheless play a positive role analogous to the formation of beneficial tribofilms through the chemical breakdown of some conventional lubricant additives like ZDDP. Thus, the ILs not only provide an adsorbed electrically addressable film, which may also have benefits for electrical applications and preventing electric discharge erosion, but they also provide a “red button” rescue function that can be operated remotely. The exigent need for such remote control is in inaccessible offshore wind and wave power installations, as well as aeronautics and space applications.

## Supplementary Information

Supplementary Information.

## Data Availability

The data underlying this article will be shared upon reasonable request to the corresponding author.
