# Supplementary material for: Tribotronic control of an ionic boundary layer in operando extends the limits of lubrication
Source: Sci Rep. 2022 Nov 28;12:20479. doi: 10.1038/s41598-022-22504-6 (PMC9705526; doi:10.1038/s41598-022-22504-6)
Supplement: Supplementary file 1 — Supplementary Information. [file 41598_2022_22504_MOESM1_ESM.pdf]

Electronic supplementary information

Tribotronic control of ionic boundary films extends in operando the limits of lubrication

Akepati Bhaskar Reddy[1], Georgia A. Pilkington[2], Mark W. Rutland[2,3,4,*], and Sergei Glavatskih[1,4,5,*]

[1]KTH Royal Institute of Technology, System and Component Design, Department of Machine Design, Stockholm, 100 44, Sweden
[2]KTH Royal Institute of Technology, Division of Surface Chemistry and Corrosion Science, Department of Chemistry, Stockholm, 100 44, Sweden
[3]Laboratoire de Tribologie et de Dynamique des Systèmes, Ecole Centrale de Lyon, Ecully, 69134, France
[4]University of New South Wales, School of Chemistry, Sydney NSW, 2052, Australia
[5]Ghent University, Department of Electromechanical, Systems and Metal Engineering, Ghent, 9052, Belgium
[*]segla@kth.se, mark@kth.se

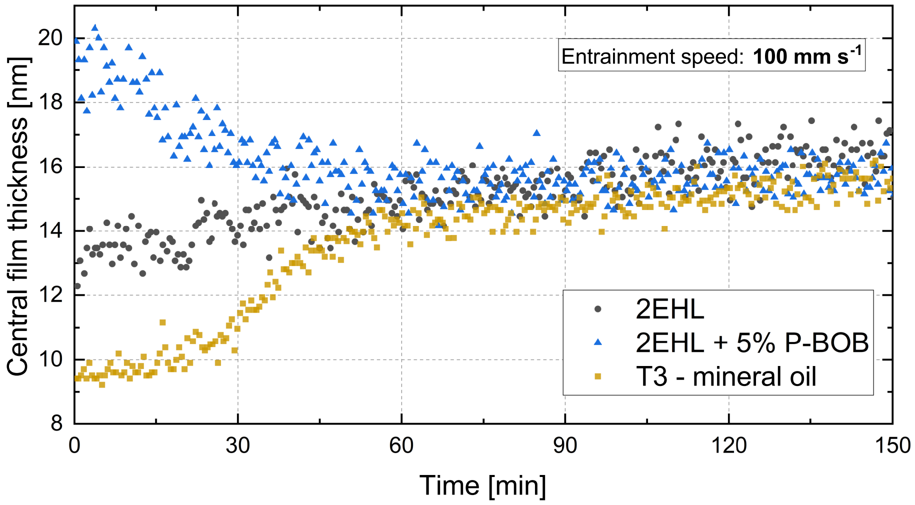

Figure S1: Open circuit central film thickness for 2EHL oil, 2EHL + 5% P-BOB solution, and T3 mineral oil as a function of time at 100 $mm\,s^{-1}$ entrainment speed.

Figure S1 shows the open circuit (OC), *i.e.* no potentials applied, time dependence of film thickness for the neat 2EHL oil, and its solution with 5% wt. P-BOB, at a constant entrainment speed of 100 $mm\,s^{-1}$. 2EHL films were observed to increase in thickness over time, which was hypothesized to be as a result of the over-rolling and packing of oil molecules on the surface. For such effects, steady-state rolling conditions are believed to be necessary without transient effects that are typically observed with speed-dependent film thickness measurements, such as acceleration, deceleration, or film squeeze effects. The oil by itself did not exhibit any electroresponsivity (Figure S2). A growth in film thickness was also observed for a non-polar mineral oil of similar viscosity (naphthenic base oil T3 from Nynas AB), albeit with a different growth trend (see Figure S1).

With the addition of 5% w/w of P-BOB to 2EHL, the film thickness trend over time changes dramatically, suggesting that the interfacial films are now dominated by the IL molecules. Initially, the film is much thicker (about 50% thicker than for oil only case), but it transitions to a thinner film comparable to the oil only case, and is moreover stable with time. The presence of P-BOB ions at the interface is also believed to prevent the formation and accumulation of the ordered oil film that was observed for the neat oil. The initial thick film, and the active domination of the ions in the boundary layer, is in agreement with the findings of the earlier study, which reported the formation of load-bearing ionic boundary films for P-BOB in 2EHL even at a low IL concentration (1 % w/w)[1]. The subsequent decrease in film thickness is believed to be due to a gradual consolidation of adsorbed ions on one or both contacting surfaces along with squeezing out of the weakly held ionic multi-layers with over-rolling. This continues until reaching a stable level, *i.e.* 16 nm of lubricating film thickness.

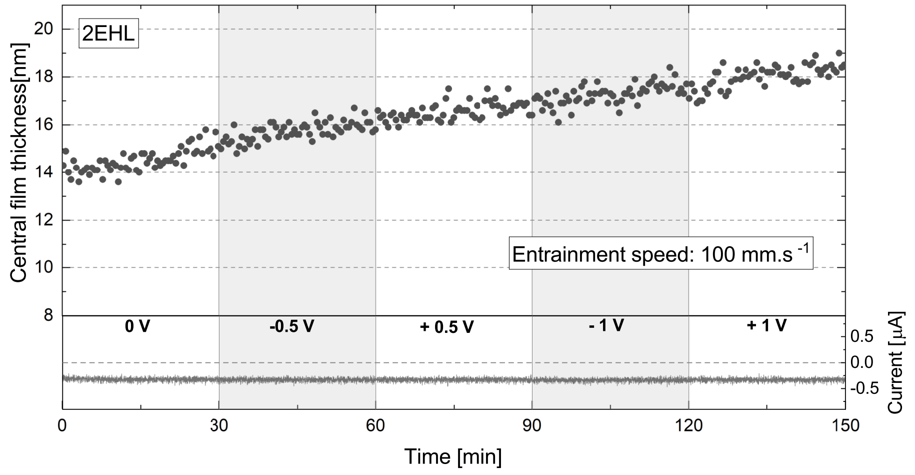

Figure S2: Central film thickness for 2EHL oil at 100 mm s^{-1} entrainment speed with applied electric potential changing every 30 minutes.

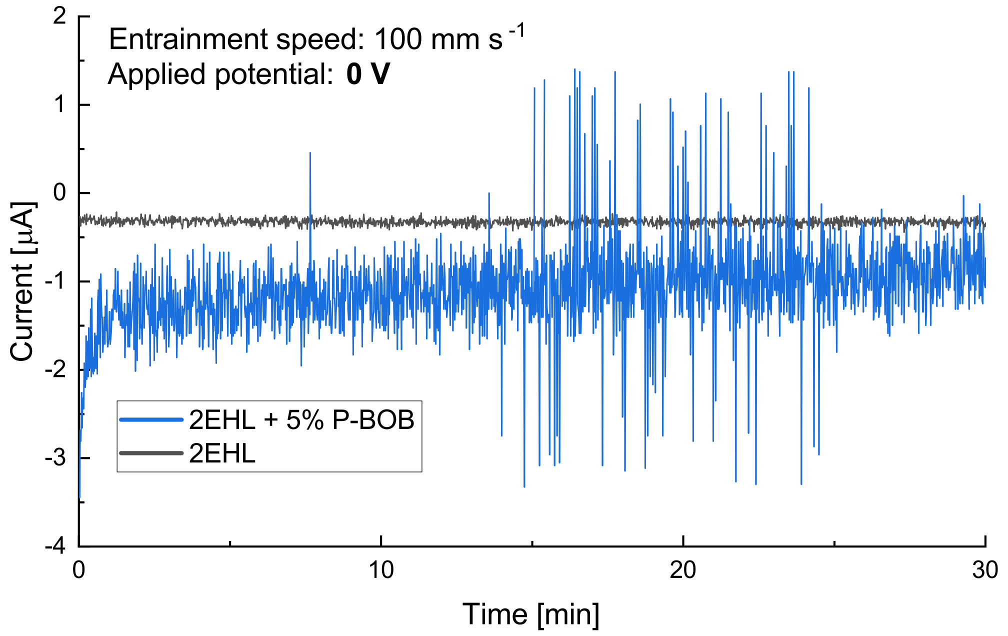

Figure S3: Electric current trace during first 30 minutes , i.e. when 0 V potential is applied.

References

[1] Reddy, A. B. *et al.* Micro- to nano- and from surface to bulk: Influence of halogen-free ionic liquid architecture and dissociation on green oil lubricity. *ACS Sustainable Chemistry & Engineering* **9**, 13606–13617, DOI: 10.1021/acssuschemeng.1c04854 (2021).
